# Supplementary material for: Three-Month Outcomes in Cancer Patients with Superficial or Deep Vein Thrombosis in the Lower Limbs: Results from the RIETE Registry
Source: Cancers (Basel). 2023 Mar 29;15(7):2034. doi: 10.3390/cancers15072034 (PMC10093050; doi:10.3390/cancers15072034)
Supplement: Supplementary file 1 [file cancers-15-02034-s001.zip › cancers-2288879-supplementary.pdf]

Coordinator of the RIETE Registry: Manuel Monreal.

RIETE Steering Committee Members: Paolo Prandoni, Benjamin Brenner and Dominique Farge Bancel.

RIETE National Coordinators: Raquel Barba (Spain), Pierpaolo Di Micco (Italy), Laurent Bertoletti (France), Sebastian Schellong (Germany), Inna Tzoran (Israel), Abilio Reis (Portugal), Marijan Bosevski (R. Macedonia), Henri Bounameaux (Switzerland), Radovan Malý (Czech Republic), Peter Verhamme (Belgium), Joseph A. Caprini (USA), Hanh My Bui (Vietnam).

RIETE Registry Coordinating Center: S & H Medical Science Service.

APPENDIX Members of the RIETE Group

SPAIN: Adarraga MD, Aibar J, Alonso J, Amado C, Arcelus JI, Ballaz A, Barba R, Barbagelata C, Barrón M, Barrón-Andrés B, Blanco-Molina A, Beddar Chaib F, Botella E, Castro J, Castro M, Chasco L, Criado J, de Ancos C, de Miguel J, del Toro J, Demelo-Rodríguez P, Díaz-Brasero AM, Díaz Pedroche MC, Díaz-Peromingo JA, Domínguez IM, Dubois-Silva A, Escribano JC, Espósito F, Exebio J, Farfán-Sedano AI, Fernández-Bouza E, Fernández-Capitán C, Fernández-Reyes JL, Fidalgo MA, Font C, Francisco I, Gabara C, Galeano-Valle F, García MA, García-Bragado F, García de Herreros M, García de la Garza R, García-Díaz C, Gil-Díaz A, Giménez-Suau M, Gómez-Cuervo C, Grau E, Guirado L, Gutiérrez J, Hernández-Blasco L, Jara-Palomares L, Jaras MJ, Jiménez D, Jiménez R, Jiménez-Alfaro C, Jou I, Joya MD, Lainez-Justo S, Latorre-Díez A, Lobo JL, López-Jiménez L, López Miguel P, López-Núñez JJ, López-Reyes R, López-Sáez JB, Lorenzo A, Madridano O, Maestre A, Manrique-Abos I, Marchena PJ, Martín del Pozo M, Martín-Martos F, Martínez-Redondo I, Martínez-Urbistondo D, Mella C, Mercado MI, Monreal M, Muñoz-Blanco A, Nieto JA, Núñez-Fernández MJ, Olid-Velilla M, Otalora S, Otero R, Parra P, Parra V, Pazos-Ferro A, Pedrajas JM, Peris ML, Porras JA, Portillo J, Rosa V, Ruiz-Artacho P, Ruiz-Giménez N, Ruiz-Ruiz J, Ruiz-Sada P, Salgueiro G, Sánchez-Martínez R, Sánchez-Muñoz-Torrero JF, Sancho T, Soler S, Suárez-Rodríguez B, Suriñach JM, Torres MI, Torres-Sánchez A, Trujillo-Santos J, Uresandi F, Valero B, Valle R, Varona JF, Vela L, Vela JR, Villalobos A, Villares P, Zamora C, **AUSTRIA:** Ay C, Nopp S, Pabinger I, **BELGIUM:** Engelen MM, Vanassche T, Verhamme P, **BRAZIL:** Yoo HHB, **CZECH REPUBLIC:** Hirmerova J, Malý R, **FRANCE:** Accassat S, Ait Abdallah N, Bertoletti L, Bura-Riviere A, Catella J, Couturaud F, Crichi B, Debourdeau P, Espitia O, Falvo N, Farge-Bancel D, Grange C, Helfer H, Lacut K, Le Mao R, Mahé I, Morange P, Moustafa F, Poenou G, Sarlon-Bartoli G, Suchon P, Quere I, **GERMANY:** Schellong S, **IRAN:** Nikandish R, **ISRAEL:** Braester A, Brenner B, Kenet G, Tzoran I, **ITALY:** Basaglia M, Bilora F, Bortoluzzi C, Brandolin B, Ciammaichella M, Corgna C, De Angelis A, Di Micco P, Imbalzano E, Mastroiacovo D, Merla S, Pesavento R, Pomero F, Prandoni P, Siniscalchi C, Tufano A, Visonà A, Vo Hong N, Zalunardo B, **LATVIA:** Kigitovica D, Skride A, Strautmane S, **PORTUGAL:** Fonseca S, Martins-Duarte F, Meireles J, **REPUBLIC OF MACEDONIA:** Bosevski M, **SWITZERLAND:** Bounameaux H, Mazzolai L, USA: Caprini JA, **VIETNAM:** Bui HM.

ACKNOWLEDGEMENTS We express our gratitude to Sanofi Spain, LEO PHARMA and ROVI for supporting this Registry with an unrestricted educational grant. We also thank the RIETE Registry Coordinating Center, S&H Medical Science Service, for their quality control data, logistic and administrative support and Prof. Salvador Ortiz, Universidad Autónoma Madrid and Silvia Galindo, both Statistical Advisors in S&H Medical Science Service for the statistical analysis of the data presented in this paper.
